# NLRP3 in the Cerebrospinal Fluid as a Potential Biomarker for the Diagnosis and Prognosis of Community-Acquired Bacterial Meningitis in Adults

**DOI:** 10.3389/fcimb.2021.803186

**Published:** 2022-01-25

**Authors:** Zhe Gong, Chaopeng Zhang, Yanfei Li, Lijun Jing, Ranran Duan, Yaobing Yao, Junfang Teng, Yanjie Jia

**Affiliations:** ^1^ Department of Neurology, The First Affiliated Hospital of Zhengzhou University, Zhengzhou, China; ^2^ Department of Neurology, The Peoples’ Hospital of Dengfeng, Dengfeng, China

**Keywords:** community-acquired bacterial meningitis, NLRP3, cerebrospinal fluid, clinical severity, prognosis

## Abstract

**Objective:**

To discover the levels of NLR family pyrin domain-containing 3 (NLRP3) in the cerebrospinal fluid (CSF) from adult patients with community-acquired bacterial meningitis (CABM).

**Methods:**

We enrolled 34 patients with CABM, 20 patients with viral meningitis (VM), and 25 patients with non-inflammatory neurological disease. Data on standard clinical parameters, scores, and outcomes were obtained from clinical records, and inflammasome levels in the CSF were measured by an enzyme-linked immunosorbent assay. The area under the receiver operating characteristic curve (AUROC) was used to quantify the diagnostic and prognostic performance of CSF NLRP3 as a biomarker of CABM.

**Results:**

The levels of NLRP3 were elevated in the CSF of patients with CABM, but levels for ASC, caspase-1, or other inflammasomes did not vary significantly. CSF NLRP3 was positively correlated with clinical severity and with the neutrophil-to-lymphocyte ratio (NLR), platelet-to-lymphocyte ratio (PLR), lymphocyte count, albumin quotient (Qalb), and immunoglobulin G quotient (QIgG). Patients with unfavorable outcomes had higher levels of NLRP3 in the CSF, which were correlated with several blood indicators, including NLR, PLR, and lymphocyte and monocyte counts.

**Conclusions:**

Our results suggested that the level of CSF NLRP3 could represent the severity of CABM in adults. CSF NLRP3 may be a good biomarker for the diagnosis of CABM and for the discrimination between CABM and VM. It may also be a better biomarker for predicting the prognosis of adult patients with CABM when compared to the NLR or the lymphocyte and monocyte counts.

## Introduction

Community-acquired bacterial meningitis (CABM) is a rare disease affecting all age groups, with an annual incidence of approximately three cases per 100,000 adults in developed countries (Bijlsma et al., 2016). Despite advances in critical care, which include early administration of antibiotics and corticosteroids, bacterial meningitis still has a high mortality rate (15%–30%) and is linked to a high risk of developing neurological deficits (Glimåker et al., 2015). The poor prognosis and neurological sequelae of severe bacterial meningitis are caused both by the infection itself and by the inflammatory response from the host (Tubiana et al., 2020). The latter involves activation of both cellular and non-cellular components of the immune system, including production of pro-inflammatory cytokines, complement-mediated leukocyte recruitment, and microglial activation (Koelman et al., 2019; Loughran et al., 2019).

The activation of toll-like receptors (TLRs) is a key event in meningeal inflammation and a pivotal factor for meningitis-associated tissue damage (Koedel, 2009). In addition, the levels of several inflammatory cytokines, such as interleukin (IL)-6, tumor necrosis factor (TNF)-α, IL-1β, and IL-18, are increased in the cerebrospinal fluid (CSF) of patients affected by bacterial meningitis (Geldhoff et al., 2013; Chong et al., 2018). Among them, IL-1β and IL-18 are the main downstream effectors of inflammasome activation (Schroder and Tschopp, 2010; Gong et al., 2018), and therefore may also be involved in the neuroinflammatory response to bacterial meningitis.

The inflammasome is a complex composed of a variety of proteins that initiate the activation of caspase-1 leading to the cleavage of the pro-inflammatory cytokines IL-1β and IL-18 (Patel, 2017). Four main types of inflammasomes have been described: nucleotide-binding leucine-rich repeat (NLR) family pyrin domain containing 1 (NLRP1), NLRP3, NLR family CARD domain containing 4 (NLRC4), and absent in melanoma 2 (AIM2) (Gross et al., 2011). The NLRP3 inflammasome is the most extensively studied but also the most elusive in central nervous system diseases (Shao et al., 2018). Previous studies found that the inflammasome proteins NLRP3 and apoptosis-associated speck-like protein containing a CARD (ASC) play important roles in the regulation of the systemic inflammatory response and development of cerebral damage during pneumococcal meningitis (Geldhoff et al., 2013). However, NLRP3 levels in the CSF from patients with bacterial meningitis have not been determined.

The aims of this study were to measure NLRP3 levels in the CSF from patients with CABM, viral meningitis (VM), and non-inflammatory neurological disease; to characterize the correlation between clinical outcomes and NLRP3 levels; and to determine whether NLRP3 levels from the CSF could be used as a diagnostic and prognostic biomarker for bacterial meningitis in clinical practice.

## Materials and Methods

### Patient Characteristics and Inclusion Criteria

This retrospective cohort study enrolled 34 patients with CABM, 20 patients with VM, and 25 patients with non-inflammatory neurological disease treated at the Department of Neurology of the First Affiliated Hospital of Zhengzhou University from June 2019 to June 2021. The study design was approved by the Zhengzhou University Ethics Committee. The inclusion criteria were as follows: (1) age ≥18 years; (2) disease first diagnosed at the Department, without previous antibacterial or anti-inflammatory treatment; and (3) confirmed bacterial meningitis by positive CSF microscopy/culture or high clinical suspicion of meningitis due to next-generation sequencing (NGS)-positive detection and increased cell count and/or decreased blood/glucose ratio in the CSF (Grønhøj et al., 2021). We excluded patients with hospital-acquired meningitis, those presenting a recent head injury (within 1 month) or who had undergone neurosurgery, those carrying neurosurgical devices, and those affected by chronic meningitis. The etiologies of CABM included ten cases (29.41%) of *Streptococcus pneumoniae*, six cases (17.65%) of *Klebsiella pneumoniae*, four cases (11.76%) of *Escherichia coli*, four cases (11.76%) of *Listeria monocytogenes*, two cases (5.88%) of *Staphylococcus aureus*, one case (2.94%) of *Bacteroides fragilis*, one case (2.94%) of human staphylococcal human subspecies, one case (2.94%) of acne propionic acid bacillus, one case (2.94%) of constellation streptococcus constellation subspecies, one case (2.94%) of *Streptococcus suis*, one case (2.94%) of *Streptococcus pharyngitis*, and one case (2.94%) of *Enterobacter cloacae*.

Patients from the VM group were diagnosed by NGS detection of viral DNA/RNA in the CSF. The etiologies of VM included eight cases of herpes simplex virus, five cases of Epstein–Barr virus, three cases of cytomegalovirus, two cases of varicella-zoster virus, and two cases of Coxsackie virus. The control group consisted of patients diagnosed with other non-inflammatory neurological diseases, including dizziness, headache, and hysteria.

### Data Collection

Data collected from all patients included medical history (hypertension, diabetes, or other co-morbidities), Glasgow Coma Scale (GCS) on admission, clinical symptoms on admission, laboratory findings on admission (CSF and blood data, and causative pathogen identity), clinical course, outcome, neurological status at discharge [determined by the Glasgow Outcome Scale (GOS)] or 3 months after onset [determined by the modified Rankin Scale (mRS) score], and treatment. Two experienced neurologists independently evaluated the CABM severity according to the GCS scores at admission, the GOS scores at discharge, and the mRS scores at follow-up. Favorable outcomes were defined as those presenting an mRS score in the 0–2 range, and unfavorable outcomes were defined as those within the 3–5 range (Rankin, 1957). All methods were carried out in accordance with relevant regulations, and all participants signed written informed consent as a prerequisite for enrolment.

### CSF Collection

CSF was collected from all patients on the date of venous blood collection. After a standard lumbar puncture, 15 ml of CSF was collected. From this sample, 10 ml was used for routine biochemical and cytological tests, NGS, *in vitro* culture, and electrophoresis tests. The remaining volume was immediately centrifuged at 4000 g for 10 min at 4°C. The supernatant was then transferred into polypropylene tubes and stored at −80°C. Sample treatment was completed within 60 min.

### Laboratory Data

All blood samples collected were tested for biomarkers at the Biochemistry Laboratory of the First Affiliated Hospital of Zhengzhou University. Fasting venous blood was drawn from all patients on the morning of the second day after admission. Blood samples were used for routine blood tests and for the measurement of inflammatory markers. For routine CSF analysis, humoral cells were counted and classified *via* instrument analysis. In parallel, the centrifuge precipitation method was used for manual microscopic examination, classification, and reexamination. The albumin quotient (Qalb) and immunoglobulin G quotient (QIgG) were detected *via* immunoelectrophoresis. CSF second-generation sequencing was performed *via* high-throughput analysis by the Beijing Institute of Genomics (Beijing, P. R. China).

### Determination of CSF Inflammasome Levels

Commercial sandwich enzyme-linked immunosorbent assay (ELISA) kits were used to measure inflammasome levels in the CSF. Target proteins included NLRP1 (CSB-EL015864HU), NLRP3 (CSB-E15885h), NLRC4 (CSB-EL015862HU), AIM2 (CSB-EL001499HU), ASC (CSB-EL019114HU), and caspase-1 (CSB-E13025h). All kits were manufactured by Cusabio (Wuhan, P. R. China). Assays were performed according to the manufacturer’s instructions. All standards and samples were measured in duplicate, and CSF samples were not diluted. Optical densities were determined using a microplate reader (Bio-Rad Laboratories, CA, USA).

### Statistical Analysis

Statistical analysis was performed using the SPSS software package (version 26.0; IBM, Armonk, NY, USA). The data were tested for normality, and normally distributed data were expressed as mean ± standard deviation. The Kruskal–Wallis test was used for comparison between groups. An independent Student’s *t*-test was used to compare data between two groups. The median (interquartile range) was used to characterize the results for non-normally distributed data, and the Mann–Whitney *U* test was used in this case to perform comparisons between groups. Categorical variables were expressed as percentage of the total number of cases and were compared using the Chi-square test or Fisher’s exact test. Univariate and multivariate analyses were used to identify the factors that could distinguish between the groups. Correlations between the profiles were assessed using Spearman’s rank analysis or Pearson’s correlation analysis, as appropriate. The area under the receiver operating characteristic curve (AUROC) was used to quantify the performance of biomarkers for a given diagnosis. The cutoff values for outcome prediction were selected on the basis of the highest sum of sensitivity and specificity. Statistical significance was set at *p* < 0.05.

## Results

### Demographics, Clinical Features, and Laboratory Data

The demographic data, clinical features, and laboratory data of CABM patients (*n* = 34), VM patients (*n* = 20), and CTLs (*n* = 25) are shown in **Table 1**. Compared to the VM cohort, fever (100%), neck stiffness (85.29%), dyssomnia (50%), and mental and behavioral disorders (44.12%) were the most common symptoms registered in the CABM group. The clinical symptom score (GCS on admission) and the prognostic score (GOS at discharge and mRS 3 months after onset) from CABM patients were significantly higher than those from VM patients.

**Table 1:** Demographics, clinical features, and laboratory data of patients.

	CABM (*n* = 34)	VM (*n* = 20)	CTL (*n* = 25)
Age (years)	41.5 ± 13.76	41.95 ± 18.14	45.596 ± 16.2
Gender (male/female)	26/8	12/8	14/11
Psychiatric and neurologic symptoms			
Headache	27/34 (79.41%)	11/20 (55%)	–
Fever	34/34 (100%)	8/20 (40%)^****^	–
Vomit	14/34 (41.18%)	7/20 (35%)	–
Neck stiffness	29/34 (85.29%)	6/20 (30%)^****^	–
Disturbance of consciousness	12/34 (35.29%)	12/20 (60%)	–
Mental and behavioral disorder	15/34 (44.12%)	2/20 (10%)^**^	–
Dyssomnia	17/34 (50%)	4/20 (20%)^*^	–
Seizures	4/34 (11.76%)	14/20 (70%)^****^	–
Cranial nerve injury	11/34 (32.35%)	2/20 (10%)	–
Weakness	7/34 (20.59%)	1/20 (5%)	–
Autonomic symptoms	6/34 (17.65%)	3/20 (15%)	–
GCS on admission [median (range)]	11.5 (3,15)	15 (11,15)^****^	–
GOS at discharge [median (range)]	5 (2,5)	5 (4,5)^***^	–
mRS (3 months after onset) [median (range)]	1 (0,5)	0 (0,1)^****^	–
Medical history			
Hypertension	5/34 (14.71%)	3/20 (15%)	–
Diabetes	4/34 (11.75%)	1/20 (5%)	–
Coronary heart disease	1/34 (2.94%)	1/20 (5%)	–
Cerebrovascular disease	5/34 (14.71%)	2/20 (10%)	–
Extracranial tumor history	1/34 (2.94%)	1/20 (5%)	–
Blood routine tests results			
WBC (×10^9^/L)	15.06 ± 7.69	9.761 ± 4.857	6.54 ± 2.174^****^
PLT (×10^9^/L)	219.9 ± 71.51	209.7 ± 58.73	243.1 ± 80.13
Neutrophil count (×10^9^/L)	13.77 ± 7.729	7.675 ± 4.622^*^	4.008 ± 1.843^****^
Lymphocyte count (×10^9^/L)	0.8644 ± 0.4822	1.502 ± 0.9193^**^	3.183 ± 6.349^****^
Monocyte count (×10^9^/L)	0.6653 ± 0.358	0.4895 ± 0.2131	0.7028 ± 1.096
NLR	22.43 ± 15.93	7.008 ± 7.687^*^	2.242 ± 1.192^****^
PLR	308.8 ± 153.2	164.3 ± 59.68^***^	136.3 ± 65.87^****^
LMR	1.905 ± 2.057	3.594 ± 2.747^**^	4.27 ± 1.605^****^
Inflammation factors			
ESR (mm/h)	28.58 ± 23.84	14.3 ± 18.61	10.74 ± 8.153^*^
CRP	82.23 ± 72.32	5.438 ± 6.551^***^	5.149 ± 10.83^****^
PCT	3,215 ± 6.908	0.07125 ± 0.06278^*^	0.09746 ± 0.1983^**^
CSF results			
CSF white blood cell count (×10^6^/L)	2485 ± 4094	26.9 ± 55.39^****^	2.76 ± 2.454^****^
CSF protein (mg/l)	1761 ± 1446	485.7 ± 326.1^**^	356.9 ± 174^****^
CSF glucose (mmol/L)	1.598 ± 1.146	3.34 ± 1.27^***^	3.726 ± 0.9806^****^
CSF chlorides (mmol/L)	121.9 ± 7.945	124.2 ± 4.515	125.7 ± 5.533^*^

Values are presented as mean ± SD, numbers, or median (interquartile range). Statistical significance in comparison with CABM group: ^*^p < 0.05, ^**^p < 0.01, ^***^p < 0.001, ^****^p < 0.0001.

GCS, Glasgow Coma Scale; GOS, Glasgow Outcome Scale; WBC, white blood cell; PLT, platelet; CSF, cerebrospinal fluid; NLR, neutrophil-to-lymphocyte ratio; ESR, erythrocyte sedimentation rate; PCT, procalcitonin.

The CABM group exhibited a significant increase in the neutrophil count, neutrophil-to-lymphocyte ratio (NLR), platelet-to-lymphocyte ratio (PLR), and levels of C-reactive protein (CRP) and procalcitonin (PCT), while at the same time exhibiting a significant decrease in the lymphocyte count and the lymphocyte-to-monocyte ratio (LMR) when compared with the other two groups (**Table 1**). The CABM group also exhibited a significant increase in the white blood cell count and protein quantity in the CSF, and a significant decrease in glucose levels relative to the VM and CTL groups (**Table 1**).

### Increased Levels of NLRP3 in CSF from Patients With CABM

**Figure 1:**
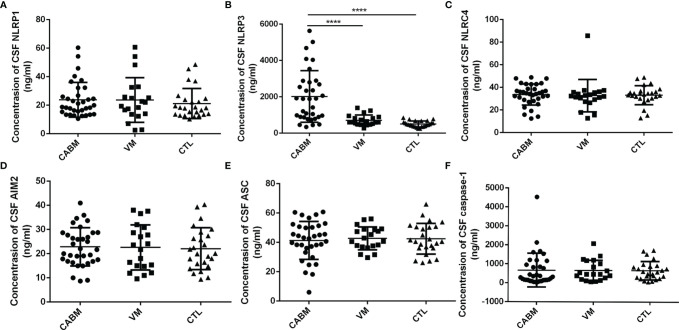
Levels of inflammasome protein in the CSF of patients with CABM. **(A, C–F)** Levels of CSF NLRP1, NLRC4, AIM2, ASC, and caspase-1 were not different among the three groups. **(B)** Concentrations of CSF NLRP3 in the CABM group were significantly higher than those in the VM (*p* < 0.001) and control (*p* < 0.001) groups. ****p < 0.001.

The results of the ELISA tests performed to measure the levels of CSF NLRP1, NLRP3, NLCR4, AIM2, ASC, and caspase-1 are shown in **Figure 1**. Mean CSF NLRP3 level (ng/ml) was 2,017 ± 1,421 for the CABM group, 709.8 ± 290.8 for the VM group, and 505.7 ± 163.9 for the control group. The values for CSF NLRP3 in the CABM group were significantly higher than those in the VM (*p* < 0.001, **Figure 1B**) and control (*p* < 0.001, **Figure 1B**) groups, but there were no significant differences between the VM and control groups (**Figure 1B**). The levels of NLRP1, NLRC4, AIM2, ASC, and caspase-1 in the CSF were not significantly different among the three groups (**Figures 1A, C–F**).

Etiological analysis showed that the most common pathogens in our study were *S. pneumoniae* (29.41%), *K. pneumoniae* (17.65%), *E. coli* (11.76%), and *L. monocytogenes* (11.76%). Mean CSF NLRP3 (ng/ml) was 2,124 ± 1,396 for *S. pneumoniae* infection, 2,606 ± 1,497 for *K. pneumoniae* infection, 2,181 ± 2,386 for *E. coli* infection, and 1,213 ± 996.9 for *L. monocytogenes* infection. However, there was no significant difference in CSF NLRP3 among different bacteria (**Supplementary Figure 1**).

**Figure 2:**
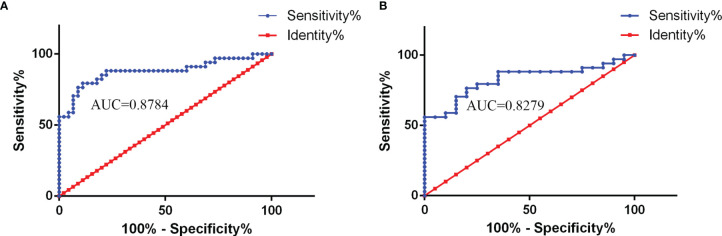
The receiver operating characteristic (ROC) curve analysis of CSF NLRP3 for the diagnosis of CABM. **(A)** NLRP3 levels showed a good area under the curve (AUC) value in the CABM vs. non-CABM comparison (AUC = 0.8784, 95% CI: 0.7932–0.9636, *p* < 0.0001). The cutoff values of CSF NLRP3 were 860.9 ng/ml with a sensitivity of 79.4% and a specificity of 88.9%. **(B)** NLRP3 showed a good area under the curve (AUC) value in the CABM vs. VM comparison (AUC = 0.8279, 95% CI: 0.7182–0.9377, *p* < 0.0001). The cutoff values of CSF NLRP3 were 892.6 ng/ml with a sensitivity of 76.47% and a specificity of 80%.

CSF NLRP3 showed good area under the curve (AUC) values for the CABM group in the receiver operating characteristic (ROC) curve analysis (CABM vs. non-CABM: AUC = 0.8784, 95% CI: 0.7932–0.9636, *p* < 0.0001; CABM vs. VM: AUC = 0.8279, 95% CI: 0.7182–0.9377, *p* < 0.0001, **Figures 2A, B**), suggesting that CSF NLRP3 may be a good diagnostic biomarker for distinguishing between CABM and VM. The cutoff values for CSF NLRP3 were 860.9 ng/ml (CABM vs. non-CABM) with a sensitivity of 79.4% and a specificity of 88.9%, and 892.6 ng/ml (CABM vs. VM) with a sensitivity of 76.47% and a specificity of 80%.

### Relationship Between CSF NLRP3, Clinical Severity, and Disease Outcome in CABM

**Figure 3:**
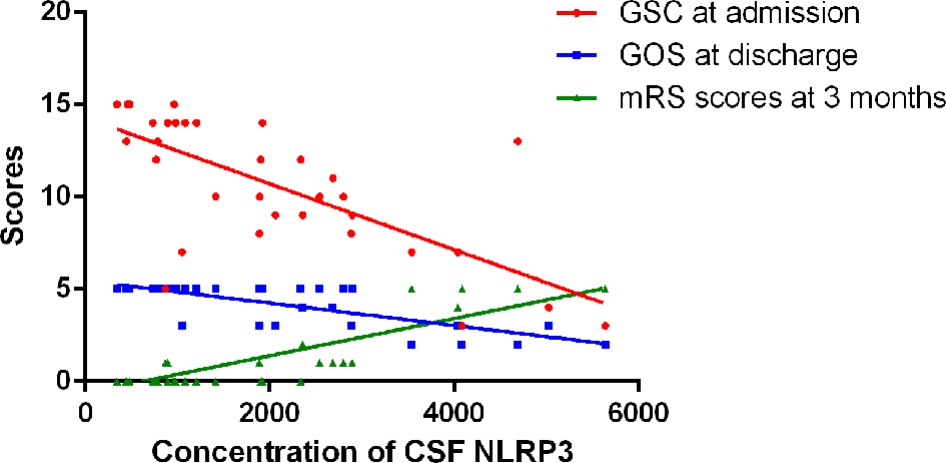
Relationships between CSF NLRP3 and clinical severity or outcome in CABM. There was a significant negative correlation between CSF NLRP3 levels and GCS scores at admission (*r* = −0.7003, *R* ^2^ = 0.4904, *p* < 0.001) or GOS scores at discharge (*r* = −0.7616, *R* ^2^ = 0.58, *p* < 0.001). There was a significant positive correlation between CSF NLRP3 levels and mRS scores 3 months after onset (*r* = 0.8052, *R* ^2^ = 0.6484, *p* < 0.001).

GCS scores at admission were used to evaluate disease severity, whereas GOS scores at discharge and mRS scores 3 months after onset were used to evaluate disease outcomes. There was a significant negative correlation in the CABM group between CSF NLRP3 levels and GCS scores at admission (*r* = −0.7003, *R* ^2^ = 0.4904, *p* < 0.001, **Figure 3**) and between CSF NLRP3 levels and GOS scores at discharge (*r* = −0.7616, *R* ^2^ = 0.58, *p* < 0.001, **Figure 3**), suggesting that patients with higher CSF NLRP3 concentrations presented with more severe disease and poorer clinical outcomes. There was also a significant positive correlation between CSF NLRP3 levels and mRS scores 3 months after onset (*r* = 0.8052, *R* ^2^ = 0.6484, *p* < 0.001, **Figure 3**).

When CABM patients were discriminated between those who presented a favorable and an unfavorable outcome, the unfavorable outcome group exhibited a significant increase in the NLR, the PLR, and the CSF NLRP3 levels, and a significant decrease in the lymphocyte and monocyte counts when compared with the favorable outcome group (**Table 2**).

**Table 2:** Laboratory data and CSF NLRP3 levels in CABM patients discriminated by prognosis.

	Favorable outcome	Unfavorable outcome
Blood routine results		
WBC (×10^9^/L)	14.97 ± 8.494	15.27 ± 5.692
PLT (×10^9^/L)	211.8 ± 74.44	239.1 ± 63.29
Neutrophil count (×10^9^/L)	13.15 ± 8.497	15.25 ± 5.572
Lymphocyte count (×10^9^/L)	1.008 ± 0.5054^**^	0.52 ± 0.1149
Monocyte count (×10^9^/L)	0.7454 ± 0.3901^*^	0.473 ± 0.1508
NLR	18.29 ± 16.16^*^	30.79 ± 12.37
PLR	239.7 ± 82.1^****^	474.8 ± 159.5
LMR	2.173 ± 2.368	1,262 ± 0.7287
Inflammation factors		
ESR (mm/h)	24.51 ± 23.23	39.86 ± 25.35
CRP	70.85 ± 67.17	109.6 ± 80.44
PCT	3.374 ± 7.342	3.025 ± 5.638
CSF results		
CSF white blood cell count (×10^6^/L)	2209 ± 4095	3148 ± 4234
CSF protein (mg/L)	1803 ± 1472	1661 ± 1453
CSF glucose (mmol/L)	1.578 ± 1.115	1.654 ± 1.321
CSF chlorides (mmol/L)	121.9 ± 7.166	121.9 ± 10.01
QALB	22.79 ± 16.17	44.42 ± 39.54
QIgG	25.16 ± 21.21	48.32 ± 35.69
IgG Index	1.188 ± 1.14	1.299 ± 1.551
intrathecal IgG synthesis	63.6 ± 73.57	108.8 ± 101.1
CSF NLRP3 (ng/ml)	1403 ± 832.8^***^	3490 ± 1489

Values are presented as the mean ± SD. Statistical significance: ^*^p < 0.05, ^**^p < 0.01, ^***^ p < 0.001, ^****^p < 0.0001.

GCS, Glasgow Coma Scale; GOS, Glasgow Outcome Scale; WBC, white blood cell; PLT, platelet; CSF, cerebrospinal fluid; NLR, neutrophil-to-lymphocyte ratio; ESR, erythrocyte sedimentation rate; PCT, procalcitonin.

**Figure 4:**
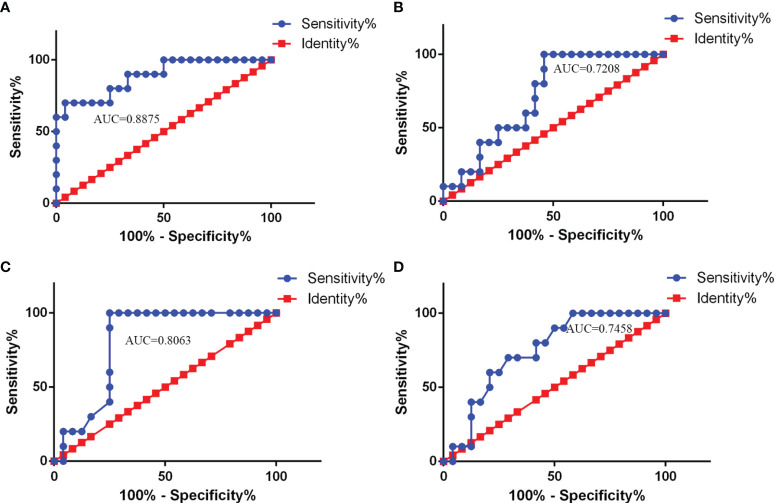
ROC curve analysis of CSF NLRP3 and other parameters used for CABM prognosis. **(A)** AUC value of CSF NLRP3 (AUC = 0.8875, 95% CI: 0.7644–1.011, *p* < 0.001) for prediction of CABM prognosis. The cutoff values were 2840 ng/ml with a sensitivity of 76.47% and a specificity of 80%. **(B)** AUC value of NLR (AUC = 0.7208, 95% CI: 0.5512–0.8905, *p* = 0.04521) for prediction of CABM prognosis. **(C)** AUC value of the lymphocyte count (AUC = 0.8063, 95% CI: 0.6587–0.9538, *p* < 0.01) for prediction of CABM prognosis. **(D)** AUC value of the monocyte count (AUC = 0.7458, 95% CI: 0.5805–0.9112, *p* = 0.02579) for prediction of CABM prognosis.

When the ROC curve analysis was performed, CSF NLRP3 levels showed a good AUC value (favorable vs. unfavorable outcome: AUC = 0.8875, 95% CI: 0.7644–1.011, *p* < 0.001), suggesting that they may also be good predictors of CABM prognosis (**Figure 4A**). In fact, CSF NLRP3 levels outperformed the NLR (AUC = 0.7208, 95% CI: 0.5512–0.8905, *p* = 0.04521, **Figure 4B**), the lymphocyte count (AUC = 0.8063, 95% CI: 0.6587–0.9538, *p* < 0.01, **Figure 4C**), and the monocyte count (AUC = 0.7458, 95% CI: 0.5805–0.9112, *p* = 0.02579, **Figure 4D**) at this task. When 2,840 ng/ml was selected as the cutoff value, CSF NLRP3 levels showed a sensitivity of 95.83% and a specificity of 70% as indicators of CABM prognosis.

### Relationship Between CSF NLRP3 and Other Parameters

**Figure 5:**
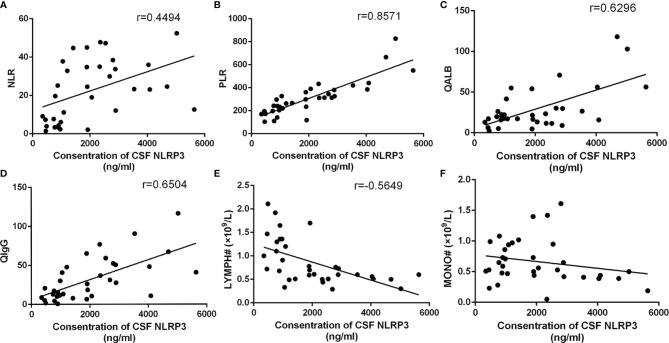
Relationship between CSF NLRP3 and other parameters. **(A)** CSF NLRP3 was positively correlated with the NLR (*r* = 0.4494, *R* ^2^ = 0.202, *p* = 0.0077). **(B)** CSF NLRP3 was positively correlated with the PLR (*r* = 0.8596, *R* ^2^ = 0.7347, *p* < 0.0001). **(C)** CSF NLRP3 was positively correlated with Qalb (*r* = 0.6296, *R* ^2^ = 0.3965, *p* < 0.0001). **(D)** CSF NLRP3 was positively correlated with QIgG (*r* = 0.6504, *R* ^2^ = 0.423, *p* < 0.0001). **(E)** CSF NLRP3 was negatively correlated with the lymphocyte count (*r* = −0.5649, *R* ^2^ = 0.3192, *p* = 0.0005). **(F)** CSF NLRP3 and the monocyte count were not correlated (*r* = −0.2203, *R* ^2^ = 0.04853, *p* = 0.2106).

Since the previous results suggested that the NLR, PLR, and lymphocyte and monocyte counts, along with CSF NLRP3 levels, were closely associated with CABM prognosis, we further explored the relevance of NLRP3 to these indicators and to some typical parameters from CSF analysis, such as Qalb and QIgG. CSF NLRP3 levels were positively correlated with the NLR (*r* = 0.4494, *R* ^2^ = 0.202, *p* = 0.0077, **Figure 5A**) and PLR (*r* = 0.8596, *R* ^2^ = 0.7347, *p* < 0.0001, **Figure 5B**), as well as with Qalb (*r* = 0.6296, *R* ^2^ = 0.3965, *p* < 0.0001, **Figure 5C**) and QIgG (*r* = 0.6504, *R* ^2^ = 0.423, *p* < 0.0001, **Figure 5D**). On the other hand, they were negatively correlated with the lymphocyte count (*r* = −0.5649, *R* ^2^ = 0.3192, *p* = 0.0005, **Figure 5E**) but not with the monocyte count (*r* = −0.2203, *R* ^2^ = 0.04853, *p* = 0.2106, **Figure 5F**).

## Discussion

In this study, we demonstrated that patients with CABM exhibit significant differences in clinical symptoms, prognostic scores, and laboratory findings when compared with VM patients, which is in accordance with previous studies (van de Beek et al., 2004; Brivet et al., 2005). In addition, our study revealed elevated levels of NLRP3 but not of ASC, caspase-1, or other inflammasomes in the CSF of patients with CABM. To the best of our knowledge, this is the first study to examine the expression levels of inflammasomes in the cerebrospinal fluid of patients with CABM.

As has been previously reported, pathogen-associated molecular patterns (PAMP) and damage-associated molecular patterns (DAMP) are the critical initial stimuli that activate NLRP3 (Hornung and Latz, 2010). The oligomerization of NLRP3 recruits ASC, which in turn cleaves pro-caspase-1 into caspase-1 (Rubartelli, 2012), inducing the release of mature pro-inflammatory cytokines, namely, IL-1β and IL-18 (Schroder and Tschopp, 2010; Gong et al., 2018). These cytokines then initiate or amplify diverse downstream signaling pathways to drive pro-inflammatory processes that may cause cellular damage, such as autophagy and pyroptosis (Schroder and Tschopp, 2010). It has been revealed that CSF levels of inflammasome-associated cytokines IL-1β and IL-18 were increased in patients with bacterial meningitis, and that this was associated with complications and unfavorable outcomes (Geldhoff et al., 2013). This piece of evidence is consistent with the increased levels of CSF NLRP3 in CABM patients that we detected. Therefore, we speculated that the NLRP3 inflammasome may play an important role in CABM. Some studies in animals have explored the role of NLRP3 in bacterial meningitis: It has been reported that the NLRP3 inflammasome contributes to brain injury in pneumococcal meningitis (Hoegen et al., 2011). In addition, *E. coli* can cause meningoencephalitis by increasing the inflammatory response and activating the TLR2/TLR4/MyD88 and the NLRP3 inflammasome pathways (Wang et al., 2020). NLRP3-deficient mice have decreased systemic inflammatory responses and bacterial outgrowth, which are also associated with an increase in cerebral neutrophil infiltration and cerebral hemorrhages (Geldhoff et al., 2013).

We also compared NLRP3 levels in CSF from CABM patients caused by different bacteria. The expression of CSF NLRP3 seemed to be increased in *K. pneumoniae* infection, but decreased in *L. monocytogenes* infection. However, due to the small sample size, there was no significant difference in CSF NLRP3 among different bacterial infections. We needed to expand the sample size for further analysis.

To date, high levels of NLRP3 in the CSF have been reported in several neuroinflammatory diseases such as anti-N-methyl-D-aspartate receptor encephalitis (Peng et al., 2019), autoimmune GFAP astrocytopathy (Luo et al., 2019), chronic inflammatory demyelinating polyradiculoneuropathy (Zhou and Xia, 2021), and neuromyelitis optica spectrum disorder (Peng et al., 2019), and are sometimes correlated with disease severity or prognosis. This strongly suggests that CSF NLRP3 has some clinical significance in neuroinflammatory diseases. In our study, there were significant positive correlations between CSF NLRP3 and clinical severity, and it was negatively correlated with GOS scores at discharge. Moreover, we found that patients with unfavorable outcomes had higher levels of CSF NLRP3 as well as higher values for several indicators measured in routine blood analysis. We sought to determine the diagnostic and prognostic performance of CSF NLRP3 as a biomarker for CABM by means of an AUROC analysis. Our results demonstrate that CSF NLRP3 may be a good biomarker for the diagnosis of CABM and for the discrimination between CABM and VM. It could also be a better biomarker for predicting the prognosis of patients with CABM compared with the NLR or with the lymphocyte or monocyte count.

Some previous studies have proposed new biomarkers for bacterial meningitis, such as serum or CSF PCT (Ko et al., 2017; Reshi et al., 2017), neutrophil-to-lymphocyte ratio (Mentis et al., 2016), persephin (Stubljar et al., 2015), heparin-binding protein (Linder et al., 2011), neurofilament (Grønhøj et al., 2021), and lipocalin 2 (Thanh et al., 2021). Due to practical limitations, only a subset of these putative biomarkers (serum PCT and NLR) were considered in the present study. Contrary to previous results, we did not find differences in the serum PCT levels among the different groups, which may have been due to the different inclusion criteria adopted in those previous studies. On the other hand, our results confirmed a significant correlation between NLR and CABM prognosis, but compared with NLR, CSF NLRP3 levels had a better predictive value.

We further analyzed the correlation between CSF NLRP3 levels and some clinical parameters. The results showed that CSF NLRP3 had a good correlation with some immune system or immune response parameters such as the NLR, the PLR, and the lymphocyte count, suggesting that CSF NLRP3 may be closely associated to the innate and adaptive immune systems. A previous study on rats (Abdul Aziz et al., 2021) had shown a correlation between NLRP3 levels in the hippocampus and the NLR. In addition, some studies have shown that the expressions of IL-1β and IL-18 in blood were correlated with the NLR or the PLR in different diseases (Zou et al., 2018; Kovács et al., 2021; Zhang et al., 2021), supporting the association between activation of the NLRP3 inflammasome and these immune response indicators. In addition, CSF NLRP3 was also clearly correlated in our study with Qalb and QIgG, indicators of blood–brain barrier (BBB) function and intracranial immune response, respectively. This strongly suggests that the high concentration of CSF NLRP3 may be closely related to the disruption of the BBB and the activation of intracranial humoral immunity. Some studies also found that the IgG index correlated with CSF IL-4 levels in patients with multiple sclerosis (Mouzaki et al., 2015).

## Limitations

Our study had some limitations. The sample size was somewhat small, and therefore the potential usefulness of CSF NLRP3 as a biomarker for CABM needs to be verified with a larger cohort. In addition, data from the blood and cerebrospinal fluid were obtained from standard hospital laboratory tests, which made us fail to further compare some newly discovered meaningful indicators in recent years. Finally, the study focused exclusively on adult patients, leaving the potential value of CSF NLRP3 as a biomarker in a pediatric context as a hypothesis to be tested in future studies.

## Conclusion

To summarize, we found substantially higher levels of NLRP3 in the CSF of adults with CABM. These levels were positively correlated with clinical severity, the NLR, the PLR, Qalb, and QIgG, and they were also negatively correlated with favorable outcomes and with the lymphocyte count, suggesting that the NLRP3 inflammasome might play an important role in the pathogenesis of CABM in adults. In addition, we found for the first time that CSF NLRP3 may be a good biomarker for the diagnosis of CABM and for the discrimination between CABM and VM cases in adults. It may also prove to be a useful potential biomarker for predicting the prognosis of individual CABM cases in adults.

## Data Availability Statement

The original contributions presented in the study are included in the article/**Supplementary Material**. Further inquiries can be directed to the corresponding author.

## Ethics Statement

The studies involving human participants were reviewed and approved by the Ethics Committee of Zhengzhou University (2019-KY-018). The patients/participants provided their written informed consent to participate in this study.

## Author Contributions

ZG carried out the experiments, performed the analysis, and wrote the manuscript. CZ contributed to the collection of the clinical data. YL and LJ contributed to data analysis. RD and YY edited the manuscript. JT collaborated with us and contributed to the design of the study. YJ designed the study and contributed to the writing and editing of the manuscript. All authors contributed to the article and approved the submitted version.

## Funding

This work was supported by the National Natural Science Foundation of China (NSFC 82001290 to ZG).

## Conflict of Interest

The authors declare that the research was conducted in the absence of any commercial or financial relationships that could be construed as a potential conflict of interest.

## Publisher’s Note

All claims expressed in this article are solely those of the authors and do not necessarily represent those of their affiliated organizations, or those of the publisher, the editors and the reviewers. Any product that may be evaluated in this article, or claim that may be made by its manufacturer, is not guaranteed or endorsed by the publisher.
